# Interprofessional learning at primary healthcare centres

**DOI:** 10.1186/s12909-026-09678-7

**Published:** 2026-06-18

**Authors:** Hanna Holst, Maria Sääf, Carina Elmqvist, Birger Pålsson

**Affiliations:** 1 Department of Health and Caring Sciences, Faculty of Health and Life Sciences, Linnaeus University, Växjö, SE-351 95 Sweden; 2 RD Kronoberg, Växjö, Sweden; 3 Research Manager for the Centre of Interprofessional Collaboration within Emergency care (CICE) Head of Research in Department of Research and Development, Region Kronoberg, Växjö, Sweden; 4 Faculty of medicine, University of Lund, Växjö, Sweden

**Keywords:** Informal care, Interprofessional education, Medical students, Nursing students, Primary Healthcare

## Abstract

**Background:**

Students can practice learning in an interprofessional environment during their education in order to enable a well-functioning cooperation between healthcare professions. The aim of interprofessional learning (IPL) is for all students to contribute with their expertise and work in teams under supervision, which has been shown to improve their understanding of their own and each other’s roles. However, previous research in interprofessional learning in primary healthcare is limited.

**Aim:**

The aim of the study was to describe and explore interprofessional learning in primary healthcare from the perspectives of patients, students, and supervisors.

**Method:**

A mixed method approach was used with 35 participants (14 students, 16 patients and 5 supervisors) in a convergent design. Data collection and the analysis of questionnaires and interviews were conducted simultaneously. The responses to the questionnaires were analysed descriptively, and the interviews were analysed thematically.

**Results:**

The results, based on the perspectives of the patients, students, and supervisors, showed that IPL supported the students in focusing on the patient and contributed to a patient-centred approach. The interprofessional learning of the students evolved gradually through an increased responsibility in the cooperation. In order to balance individual and collective learning, the supervisors could take a step back to support the students’ pursuit of independence.

**Conclusion(s):**

These findings suggest that IPL could improve and develop quality in primary healthcare. The results of the present study emphasize that the patient perspective plays an important role in students’ interprofessional learning as it contributes to a holistic approach and perspective in healthcare. The supervisors play an important role in structuring the IPL, acting as role models, nurturing cooperation between the students and being responsible for patients’ safety.

**Supplementary Information:**

The online version contains supplementary material available at 10.1186/s12909-026-09678-7.

## Background

The World Health Organization’s definition of interprofessional learning is when two or more professions learn from, with, and about each other [1]. The aim of Interprofessional learning (IPL) is for all students to contribute with their expertise and work in teams under supervision, which has been shown to improve their understanding of their own and each other’s roles [2]. Students can practice learning in an interprofessional environment during their education in order to enable a well-functioning cooperation between healthcare professions. The development of learning strategies during clinical educations should provide students with an opportunity to prepare for their professional lives. Previous research [3] shows that students who cooperate interprofessionally during their education also cooperate interprofessionally as professionals. IPL contributed to a more holistic view of patient situations, and that students learning together in IPL complemented each other, realizing the need for different roles and understanding that no one could do everything alone [4].

Primary healthcare is the healthcare system, closest to the population, providing outpatient care for common needs. A patient-centered approach was found in primary healthcare, the patient could become the teacher who optimized care and improved their health in the learning environment when students from different programs provided care together under supervision. Patients were encouraged to have more involvement in decisions regarding their own health, and students focused on treating the patient as an equal in the interprofessional team. The students reflected on what went well and what they could have done better for their own development after the appointments and also described the supervisors as creating an exceptional environment for learning [5]. Previous research [6] also shows that it is important to invite the patients to participate in the students’ learning process, as well as their own health process in the interactions between students and patients. Patients usually want to support students and contribute to their learning and future profession through the positive atmosphere that is created.

The supervisor also plays an important role in helping the patient feel comfortable and secure in these situations. The interaction between the patient, the student, and the supervisor is crucial for the well-being of the patient. The student’s and supervisor’s knowledge in the context of both learning and caring contributes to a sense of security for the patient [6]. Previous research [7] maintain that the sharing of ideas among students and supervisors promotes learning, and that the involvement of the patient was appreciated by everyone in the clinical work, as well as the diverse backgrounds of the team, which fostered collegiality.

The competence of healthcare professionals requires continuous learning and an exchange of experiences as primary healthcare centres have been given a changed and expanded assignment. An important part of this is to utilize and develop interprofessional attitudes and learning environments [8]. However, previous research [9] in interprofessional learning in primary healthcare is limited.

## Aim

The aim of the study was to describe and explore interprofessional learning in primary healthcare from the perspectives of patients, students, and supervisors.

### Method

The study was conducted using a mixed method approach in a convergent single phase. This method is suitable for use in under-researched fields where the research question guides the study [10]. The research questions were, *how can interprofessional learning take place in collaboration with patients cared for in primary healthcare centres?* and *How can learning support for interprofessional learning be developed?*

**Fig. 1:**
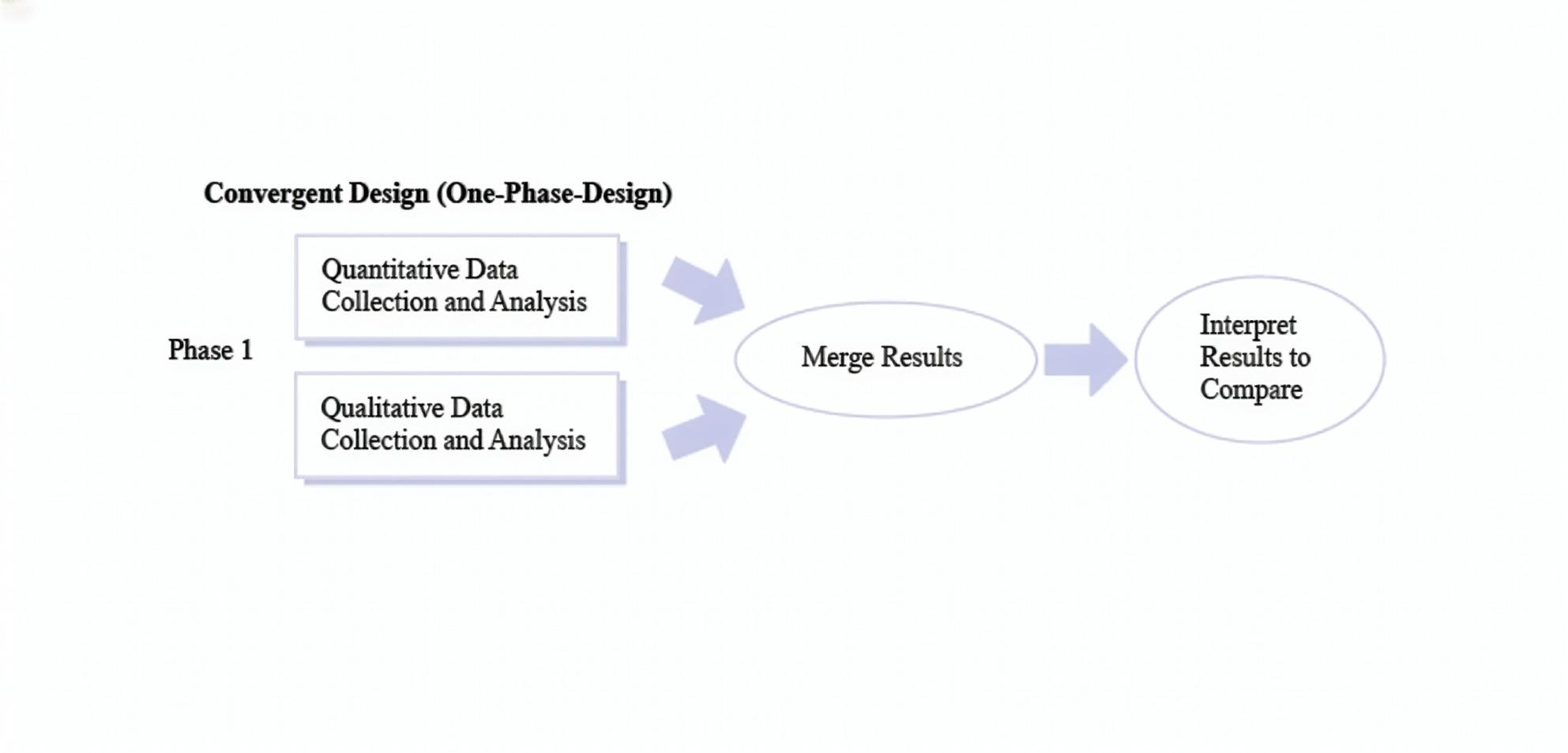
Convergent Design

Consideration and attention were given by the research group (authors) as to how the quantitative and qualitative components were related to each other and how they could be presented. The convergent single phase combined the qualitative and quantitative approaches for a deeper analysis, which in turn provided a more comprehensive understanding of the findings and the ability to generalize the results to a larger population (Fig. 1). Questionnaires and interviews are valued equally in the present study [10].

### Context

The project was carried out at two healthcare centers in a regional healthcare service in southern Sweden. Healthcare professionals work in teams at Swedish primary healthcare centres to provide general medical care, preventive services, and support for both physical and mental health. Medical students practice at the healthcare centres during their 11th and final term and nursing students during their 6th and final term.

A medical student and a nursing student learned and cared for four patients per day together. The students planned, organized and cared for the patients together with a reflective support from their supervisors before, during and after each meeting. The medical and nursing supervisors supported the students in their cooperation. The study included ten nursing students, four medical students, 16 patients and four nursing supervisors and one doctoral supervisor.

### Inclusion criteria

Patients, 18 years or older, who could express their experiences of being cared for by students engaged in interprofessional learning and whose current condition was not negatively affected by participation were included. The selection of students included those who were placed at each primary healthcare center as well as the supervisors for each profession.

### Sampling

Patients were recruited continuously and received information at the same time as they booked their appointment. The students and the supervisors received information before the start of the students’ clinical practice. The selection was based on voluntary participation. All the respondents received information letters and had the opportunity to ask questions and consider their participation before signing a consent form.

### Data collection

Data is based on 35 questionnaires and interviews with all the participants (Table 1). All of them completed the questionnaire (first) and were interviewed (second) at the same time. The data collection started in the autumn of 2022 and continued until the end of 2023. The patients were interviewed during their healthcare appointments, and the students at the end of their clinical placement period, and the supervisors after supervising students on at least two occasions.

**Table 1:** Overview of mixed method according to convergent design in one phase used for data analysis in the present study [10]

PHASE 1 DESIGN (Convergent design)	DATA MANAGEMENT	PRODUCT
Quantitative Data Collection	Questionnaire (*n* = 35)	Numerical Data
Quantitative Data Analysis	Frequence Percentage	Descriptive Statistics
Qualitative Data Collection	Interview (*n* = 35)	Text Data
Qualitative Data Analysis	Thematic Analysis	Codes Categories
Merging of results, of Quantitative and Qualitative Data	Interpretation of Quantitative and Qualitative results for comparison	Synthesis

The research group designed the questionnaires (supplementary file) based on the knowledge of themes and topics developed from a scoping review [9]. They consisted of 7 statements for the patients (Table 2), 17 statements for the students (Table 3), and 18 statements for the supervisors (Table 4). The responses were made on a four-point Likert scale to reduce complexity. The response options ranged from 1 = completely agree, 2 = mostly agree 3 = slightly agree, to 4 = do not agree at all.

**Table 2:** Overview of the answers from the patients. Patients’ perspective *n* = 16

Number	Statement	Proportion of patients who had a high level of agreement with the statement	Nr, of responses
1	The students listened to me when I spoke about my health and my experiences of problems and difficulties	100%	16
2	The students were committed in their care for me	100%	16
3	I felt secure in the care meeting	100%	16
4	I encouraged the students in their cooperation	81%	16
5	The students worked well together	100%	16
6	The students could answer my questions	94%	16
7	I received the same answer from both students	94%	16

**Table 3:** Overview of the answers from the students. Students’ perspective *n* = 14

Number	Statements	Proportion of students who had a high level of agreement with the statement	Nr. of responses
1	We listened to the patients when the different professions cooperated	100%	14
2	I knew which resources were available to care for the patient together with my student colleague	100%	14
3	The cooperation with students from other professions contributed to a personalised care for the patients	93%	14
4	We, the students, invited each other in to cooperate in the care of the patient in order to gain more knowledge and a greater understanding of the patients’ situation	100%	14
5	The students from different professions planned the patients’ care together	79%	14
6	I experienced that reflecting together with students from other professions supported me in my work	86%	14
7	The cooperation with students from other professions contributed to a sense of security	93%	14
8	The cooperation with students gave greater understanding and knowledge about each other’s professions	93%	14
9	I am positive about cooperating with other professions in my future work	100%	14
10	There were factors that obstructed learning between students from different professions	27%	14
11	I experienced support from my supervisor(s) when reflecting	100%	14
12	There were opportunities to put questions to the supervisors during the cooperation between the professions	100%	14
13	The supervisors have been good role models in the cooperation between the professions	100%	14
14	The supervisors had a positive attitude to learning between the professions	100%	14
15	I learned by discussing with other professions when we cared for patients together	100%	14
16	I experienced that the care was patient safe when we cared for patients together	100%	14
17	I would like to have more learning between professions during clinical training	93%	14

**Table 4:** Overview of the answers from the supervisors. Supervisors’ perspective *n* = 5

Number	Statements	Proportion of supervisors who had a high level of agreement with the statement	Nr. of responses
1	I could support the students in their learning about caring for patients together - with a focus on patients’ needs	100%	5
2	When the students from different professions have cared together. it has contributed to a personalised care for the patients	80%	5
3	The students planned and discussed with each other how they were going to care for the patients.	80%	5
4	The students invited each other in to cooperate in the care of the patient in order to gain more knowledge and a greater understanding of the patients’ situation	60%	5
5	The students gave the same answers to the patients	80%	5
6	There were factors that obstructed learning between students from different professions	60%	5
7	Sufficient time for the students’ learning between the professions was allowed	100%	5
8	I see myself as a role model for the students’ possibilities for learning from other professions	80%	5
9	The students’ learning between professions has contributed to greater knowledge among the staff	20%	5
10	There is time for the students to get to know each other	20%	5
11	I could support the students in their learning to care for patients together - with a focus on the learning environment and the supervisory role	60%	5
12	There was sufficient space for the students’ learning between the professions	60%	5
13	Reflection together with colleagues and students from different professions has been carried out and has contributed to greater knowledge	60%	5
14	I have a positive attitude to students’ learning between professions	80%	5
15	There are opportunities for the students to put questions to us as supervisors during the cooperation between the professions	80%	5
16	I experienced that that the care was patient safe when the students from different professions cared for the patients together	80%	5
17	Learning between professions generated new approaches to the care of patients that contributed to new courses of action and improvements in the care of the patients	60%	5
18	I experienced that the collaboration was good between the university and the clinic	80%	5

The interviews were conducted [11] and started with an open-ended question, such as asking the patients “*how did you experience being cared for by students from different professions learning together?*“. The purpose of the initial interview question was to be open and responsive to the informant’s experience. This entailed the researcher adopting a reflective attitude characterized by following the informant’s experiences of interprofessional caring and learning in primary healthcare and asking follow-up questions based on what the informant said. The interviews lasted for 15–45 min. The data collected from the interviews were recorded and transcribed verbatim to ensure the accuracy of the content.

### Data analysis

The data analysis of the responses to the questionnaires was carried out according to Creswell’s single-phase approach [10]. For descriptive statistics, percentages calculated for the levels of agreement by reducing the Likert scores of 1 and 2 to a ‘high level of agreement’. The data collected from the questionnaires were analysed separately for each perspective in SPSS 29.0.2.0 and presented descriptively.

The data from the interviews were read multiple times to enhance familiarity with the material and deepen the understanding of the participants’ experiences and were subsequently analysed using a reflective thematic approach [11]. Meaning units that addressed the aim were identified and marked with a temporary code. The search for patterns of meanings led to the creation of overarching themes from the codes that described similar aspects of the aim. The themes at the end of the analysis were named based on what was described in each theme, *Time and more profound communication*,* Learning together* and *Safety in supervision* [11].

The integration of the qualitative and quantitative findings was made possible through the interpretation and comparison of the results from the respective data collections being as these were based on the same research aim. This process revealed similarities and differences that could be synthesized into a new, overarching result.

## Results

### Results from the questionnaires

The sixteen patients generally responded with high levels of agreement with the seven statements, as shown in Table 2. The patients responded similarly concerning the students’ ability to answer their questions and that they received consistent answers from the two students. Finally, 81% responded positively to the statement concerning giving encouragement to the students in their cooperation.

The fourteen students had a high level of agreement with 10 of the 17 statements, as illustrated in Table 3. All but one of them had a high level of agreement with four of the statements: cooperation with students from other professions contributed to individualized care for the patients, to a sense of security, provided a greater understanding and knowledge of each other’s professions and desiring more IPL between the professions during clinical practice. Twelve of the students felt that reflecting together with students from other professions provided support in their work, while eleven agreed that planning around the patient’s care was carried out jointly by students from different professions. On the other hand, only four of them agreed to there being hindering factors for learning between students from different professions.

The five supervisors responded to 18 statements (Table 4). There were high levels of agreement with ten of the statements, while there was 60% agreement with six of the statements. There were significantly lower levels of agreement (20%) with two of the statements - interprofessional learning among students has contributed to increased knowledge within the staff group and there is time for the students to get to know each other.

### Qualitative results

#### Time and more profound communication

The patients perceived that the students were prepared and thorough, and that they complemented each other based on their professional roles. The dialogue was perceived as positive and they received similar responses from the students, which contributed to the patients feeling listened to and included in the conversation. The cooperation between the patient and the students made them feel involved and the students were perceived as professional. One patient said: ” We did it really well, this conversation was very professional because one wants a professional approach, but at the same time a human approach, I can say that. I like that” The students showed genuine interest and concern for the patients’ needs and by asking appropriate questions, the patient felt validated and listened to. The supervisors were seen as role models and contributed to creating balance in the encounter between patient and student.

#### Learning together

The students found it enlightening to share thoughts and listen to each other before meeting the patient to gain a common view. A mutual respect developed between the students, and they appreciated working together in pairs. They felt that they listened to each other, learned from each other and could complement each other’s skills. One student said ”It became more profound being as it was both about the medical aspects and yes, but also everything around the patient, so I thought that we got an overall picture …” The relationship helped patients open up and a mutual dialogue was created, where everyone in the care meeting was perceived as active participants. The students felt that the supervisors contributed to a safe and educational atmosphere where it was important for the students that there was an opportunity for reflection.

#### Safety in supervision

The supervisors believed that the students’ interprofessional learning was dynamic and that the students became confident in their cooperation by planning together and in the care meeting with the patient. One supervisor said: ”they were so certain and secure in themselves. It felt safe to leave the responsibility to them …”The students’ cooperation and relationship increased their understanding and learning about each other’s professions, which in turn was perceived to contribute to improved quality of care and patient safety. The supervisors planned and showed interest in the students’ learning opportunities by showing the way and acting as a bridge between the students. The IPL project was considered rewarding, however, there were improvements to be made regarding the structure, including student scheduling, which was controlled by two universities.

### Interpretation of the findings from the two methodologies

#### Learning together

The interprofessional learning among students in primary healthcare involved shared preparations and engagement, where the students had solely positive experiences of learning with a student from another profession. The positive cooperation contributed to the integration and complementarity of their professional roles, as well as a sense of security in patient encounters, where a large majority of the patients felt they received a unified response from the students. All but one of the supervisors reported agreed that the students’ interprofessional learning was dynamic and that the students became confident in their cooperation by planning together and in the care meeting with the patient.

Learning together generated increased self-confidence, mutual respect, and was perceived to promote patient safety in the care. The structured cooperation helped reduce hierarchical barriers. The interprofessional learning of the students evolved gradually through increased responsibility in the cooperation. It was described as dynamic, and the deeper relationships between the students ensured they gained confidence and enriched the patient care. The supervisors contributed with new perspectives when solving different problems, and all of the supervisors thought they supported the new knowledge that emerged from the interaction between students and patients.

#### Time and more profound communication

All of the supervisors thought that there was sufficient time in the healthcare encounter, which contributed to all of the patients perceived they were seen and listened to. The communication occurred with mutual respect, creating a trusting relationship when students explained in a way that the patient understood. The deepened healthcare encounter in which the patients’ needs were focused on became possible when there was time to listen to the patient’s story.

The students’ interprofessional learning contributed to new perspectives in the interaction with patients by collectively taking responsibility for the patient as nearly all of the students reported that they learned from and with each other. The students reported that all the supervisors provided time and support and that the conditions for collaborative learning improved as well as the understanding of the patient’s healthcare needs and wellbeing. There was total agreement concerning good relationships between the students, which contributed to a caring relationship with the patients being created through the students’ interprofessional learning from each other, and the conception of a holistic view of the patient.

Communication and cooperation among students improved when supervisors took a step back and maintained a balance between the students’ need for independence and support. Opportunities for reflection between supervisors and students provided possibilities for feedback and learning between the professions, contributing to an understanding of the areas of responsibility and knowledge of each professions in the interaction with patients.

#### Safety in supervision

The supervisors contributed to a sense of safety for the students and patients in the caring encounter, even if they were not present in the room but were available as support. The supervisors created an educational atmosphere where students received support in a permitting climate. The students agreed completely about the support they received from the supervisors, which contributed to the creation of a safe and educational atmosphere where it was important for the students that there was an opportunity for reflection. The supervisors guided and posed open and reflective questions that contributed to a favorable learning and collaborative environment.

The administration of the students’ clinical training was impeded by the involvement of two educational institutions, and the lack of available placements and scheduling became a major challenge. However, a majority of the supervisors agreed that the cooperation with the universities had been good.

## Discussion

The aim was to describe and explore interprofessional learning in primary healthcare from the perspectives of patients, students, and supervisors. The interpretation of the results in the light of previous research is discussed first and then followed by a discussion of the strengths and weaknesses of the methodology.

### Discussion of results

The students’ interprofessional learning in primary healthcare contributed to a deepening of relationships between students and generated mutual respect, which promoted a sense of security. The results emphasized that this contributed to an increase in self-confidence as their professional roles intertwined and the care they provided felt safe for patients. This concurs with the findings of other studies, where interprofessional learning among students aims to develop knowledge and understanding of each other’s professions and competencies while becoming aware of one’s own [9].

Further, the results of our study showed that interprofessional learning promotes a holistic approach and perspective in healthcare, which helps in the development of patient-centered care in the encounter with the patient. The results also includes the patient perspective of students interprofessional learning and showed that patients felt safe when they were listened to, which created participation and involvement in the care provided. This is in line with previous research [12], who showed that patients with complex problems who required cooperation between professions naturally led to the use of different competencies when learning interprofessionally. This was confirmed in previous research [13], where patients emphasized that they were treated respectfully and taken seriously, and that a calm and friendly manner made them feel included and understood.

The current study also showed that developing structures for learning models contributed to increased opportunities for interprofessional cooperation between both students and supervisors. Similarly, positive effects of interprofessional learning are also shown in previous research within inpatient care [2, 14, 15]. The positive results could be compared with those in previous research [12, 16], who observed hierarchies between different professions that held back students and hindered them from cooperating, communicating, and sharing knowledge with each other. They found that introducing interprofessional learning (IPL) during education made it easier for students to establish contact and be motivated to continue cooperating interprofessionally after graduation. Previous research [17] observed that prejudices among professionals diminished as a sense of security increased, while curiosity and openness toward new perspectives, as well as cooperation, proved beneficial for the learning process. Furthermore, previous research [18] highlighted the importance of ensuring that all professional groups within primary care are afforded the opportunity to engage in training grounded in interprofessional learning. Previous research [12] also found that the supervisors were seen as role models, and the students needed to see how they cooperated with other professions at the healthcare center, which helped break down hierarchies.

However, previous research [4, 9, 13] found that it is also found important for students to increase their independence and competence in an environment that stimulates cooperation and learning. This, in turn, motivates students for interprofessional learning after graduation, as shown in a previous study [4], in which it was also observed that students preferred interprofessional learning to take place early in their education. The importance of cooperation between primary care centers, regional health authorities, and higher education institutions was emphasized as a prerequisite for achieving success in interprofessional learning within primary care [19].

The findings from this study, together with previous research, highlight the importance of generating relationships between students from different professions, supervisors, and patients. These relationships are significant not only for learning and caregiving, but also for the development of the students’ professional identity. The results suggest that interprofessional learning during education is valuable for future professional practice, as students who engage in such cooperation are more likely to continue working interprofessionally. Furthermore, interprofessional cooperation in both education and professional life may enhance the likelihood of patients receiving holistic care, as multiple professional perspectives are integrated into the care process. This suggests that IPL could improve and develop the quality of healthcare, as argued by previous research [20].

### Methodological discussion

The questionnaires were developed by the research group, using the headings from a literature review [9]. One limitation of the written statements is that they were not tested prior to the study, which may have resulted in some being perceived as unclear or ambiguous; however, the author has extensive experience in similar contexts, and the credibility of the work was further supported through review by critical colleagues. The statements in the questionnaires were partly the same as those used in the interviews, which might strengthen the validity, as well as the fact that the informants responded to all the statements.

The risk of “selection bias” should also be pointed out, i.e. there may be a preponderance of informants who initially have a positive attitude [10]. One medical student supervisor, who participated in the project, dropped out at the end of the project, due to a change of workplace, which resulted in only one medical student supervisor being included. This may have affected the depth of the results from the supervisor group. However, as a group, the statements from the various supervisors did not diverge significantly and can therefore be considered to be representative of the supervisor group as a whole [10]. The researchers strived to restrain their understanding of the aim being studied during the interview by allowing the informants’ experiences to be the focus and guide the direction of the interview.

The reliability of the study is strengthened when data from the quantitative and qualitative material are combined, thus contributing different information and revealing patterns. The use of similar statements in the questionnaires and questions in the interviews made it possible for comparisons to be drawn between the two data sources, which provided a deeper understanding and a nuanced picture of the results. Quotations have been included in the analysis from all three perspectives of the qualitative data in order to exemplify and provide an interpretation of the results, as well as strengthen claims and credibility [11, 21].

### Conclusion, implications and suggestions for further research

The findings suggest that the students’ interprofessional learning contributed to new perspectives in the interaction with patients by collectively taking responsibility for the patient as they learned from and with each other. The supervisors play an important role in structuring the IPL, acting as role models, creating opportunities for cooperation between the students and being responsible for patients’ safety.

There is a need to gain a greater understanding of IPL in primary healthcare, both in terms of strengthening the evidence base and of gaining insight into the long-term impact, and the following research directions are proposed to meet these needs:

- Future research should include a larger number of participants and representatives from a broader range of professions, such as occupational therapists, psychologists, and dietitians. This would allow for a more nuanced analysis of how IPL is experienced and its impact across different professional roles.
- Studies with longer follow-up periods are needed to capture the long-term effects of IPL on both patients, students and supervisors. A longitudinal design can provide valuable insights into how attitudes, cooperation skills, and professional identity evolve over time.

## Supplementary Information

Supplementary Material 1.

Supplementary Material 2.

## Data Availability

The data that support the findings of this study are available from Region Kronoberg but restrictions apply to the availability of these data, which were used under license for the current study, and so are not publicly available. Data are however available from the authors upon reasonable request and with permission of Region Kronoberg.
