# Supplementary material for: Interprofessional learning at primary healthcare centres
Source: BMC Med Educ. 2026 Jun 18;26:1004. doi: 10.1186/s12909-026-09678-7 (PMC13281429; doi:10.1186/s12909-026-09678-7)
Supplement: Supplementary file 1 — Supplementary Material 1. [file 12909_2026_9678_MOESM1_ESM.docx]

**Interview guide – patient perspective: interprofessional learning in primary health care**

**Open introductory question**

How did you experience being cared for by students who were learning together across different professions?

**Interview guide – student perspective: interprofessional learning in primary health care**

**Open introductory question**

Could you describe a day when you provide patient care together with a student from another profession?

**Interview guide – supervising perspective: interprofessional learning in primary health care**

**Open introductory question**

Could you describe a day when you supervised students who were learning together across different professions?

**Examples of follow-up questions based on what the informant shares**

Would you like to tell med more about that?

Could you describe this further?

What were your thoughts at that moment?

What did you feel at that time?
