# Supplementary material for: Interprofessional learning at primary healthcare centres
Source: BMC Med Educ. 2026 Jun 18;26:1004. doi: 10.1186/s12909-026-09678-7 (PMC13281429; doi:10.1186/s12909-026-09678-7)
Supplement: Supplementary file 2 — Supplementary Material 2. [file 12909_2026_9678_MOESM2_ESM.docx]

Questionnaire for patients

| Number | Statement |
| --- | --- |
| 1 | The students listened to me when I spoke about my health and my experiences of problems and difficulties  1 2 3 4 |
| 2 | The students were committed in their care for me  1 2 3 4 |
| 3 | I felt secure in the care meeting  1 2 3 4 |
| 4 | I encouraged the students in their cooperation  1 2 3 4 |
| 5 | The students worked well together  1 2 3 4 |
| 6 | The students could answer my questions  1 2 3 4 |
| 7 | I received the same answer from both students  1 2 3 4 |

Questionnaire for students

| Number | Statements |
| --- | --- |
| 1 | We listened to the patients when the different professions cooperated  1 2 3 4 |
| 2 | I knew which resources were available to care for the patient together with my student colleague  1 2 3 4 |
| 3 | The cooperation with students from other professions contributed to a personalised care for the patients  1 2 3 4 |
| 4 | We, the students, invited each other in to cooperate in the care of the patient in order to gain more knowledge and a greater understanding of the patients’ situation  1 2 3 4 |
| 5 | The students from different professions planned the patients’ care together  1 2 3 4 |
| 6 | I experienced that reflecting together with students from other professions supported me in my work  1 2 3 4 |
| 7 | The cooperation with students from other professions contributed to a sense of security  1 2 3 4 |
| 8 | The cooperation with students gave greater understanding and knowledge about each other’s professions  1 2 3 4 |
| 9 | I am positive about cooperating with other professions in my future work  1 2 3 4 |
| 10 | There were factors that obstructed learning between students from different professions  1 2 3 4 |
| 11 | I experienced support from my supervisor(s) when reflecting  1 2 3 4 |
| 12 | There were opportunities to put questions to the supervisors during the cooperation between the professions  1 2 3 4 |
| 13 | The supervisors have been good role models in the cooperation between the professions  1 2 3 4 |
| 14 | The supervisors had a positive attitude to learning between the professions  1 2 3 4 |
| 15 | I learned by discussing with other professions when we cared for patients together  1 2 3 4 |
| 16 | I experienced that the care was patient safe when we cared for patients together  1 2 3 4 |
| 17 | I would like to have more learning between professions during clinical training  1 2 3 4 |

Questionnaire for supervisors

| Number | Statements |
| --- | --- |
| 1 | I could support the students in their learning about caring for patients together  1 2 3 4 |
| 2 | When the students from different professions have cared together. it has contributed to a personalised care for the patients  1 2 3 4 |
| 3 | The students planned and discussed with each other how they were going to care for the patients.  1 2 3 4 |
| 4 | The students invited each other in to cooperate in the care of the patient in order to gain more knowledge and a greater understanding of the patients’ situation  1 2 3 4 |
| 5 | The students gave the same answers to the patients  1 2 3 4 |
| 6 | There were factors that obstructed learning between students from different professions  1 2 3 4 |
| 7 | Sufficient time for the students’ learning between the professions was allowed  1 2 3 4 |
| 8 | I see myself as a role model for the students’ possibilities for learning from other professions  1 2 3 4 |
| 9 | The students’ learning between professions has contributed to greater knowledge among the staff  1 2 3 4 |
| 10 | There is time for the students to get to know each other  1 2 3 4 |
| 11 | I could support the students in their learning to care for patients together  1 2 3 4 |
| 12 | There was sufficient space for the students’ learning between the professions  1 2 3 4 |
| 13 | Reflection together with colleagues and students from different professions has been carried out and has contributed to greater knowledge  1 2 3 4 |
| 14 | I have a positive attitude to students’ learning between professions  1 2 3 4 |
| 15 | There are opportunities for the students to put questions to us as supervisors during the cooperation between the professions  1 2 3 4 |
| 16 | I experienced that that the care was patient safe when the students from different professions cared for the patients together  1 2 3 4 |
| 17 | Learning between professions generated new approaches to the care of patients that contributed to new courses of action and improvements in the care of the patients  1 2 3 4 |
| 18 | I experienced that the collaboration was good between the university and the clinic  1 2 3 4 |
